# Intramural Ectopic Pregnancy Following Myomectomy

**DOI:** 10.1177/2324709618790605

**Published:** 2018-07-22

**Authors:** Deanne Vagg, Lima Arsala, Shamitha Kathurusinghe, W. Catarina Ang

**Affiliations:** 1 Royal Women’s Hospital, Parkville, Victoria, Australia

**Keywords:** intramural ectopic pregnancy, myomectomy, transvaginal ultrasound

## Abstract

Intramural pregnancy is a rare form of ectopic pregnancy with early diagnosis essential for prevention of severe hemorrhage and uterine rupture. We report a rare case of an intramural ectopic pregnancy at 12 weeks gestation in a woman 1 year post open myomectomy. Both transvaginal ultrasound and magnetic resonance imaging were utilized as diagnostic aids in this case. The rare nature of this clinical scenario and lack of guidelines for management made clinical decision making difficult. Due to the size and location of the gestational sac, hysterectomy was deemed to be the safest modality, and a midline laparotomy, total abdominal hysterectomy, and bilateral salpingectomy was performed.

## Introduction

Intramural ectopic pregnancy is described as a pregnancy that is partially or completely located within the myometrium of the uterine wall, without connection to the fallopian tubes or endometrial cavity.^1^ It is characterized by trophoblastic invasion that extends beyond the endometrial-myometrial junction, with invasion into the myometrium.^2^ Diagnosis requires visualization of trophoblastic invasion into the myometrium, most commonly performed with transvaginal ultrasound or magnetic resonance imaging (MRI).^3^ Intramural pregnancy is a rare diagnosis, accounting for less than 1% of all ectopic pregnancies.^4^

There is limited evidence to guide management of intramural ectopic pregnancy. Medical treatment involves using localized methotrexate with or without potassium chloride and systemic methotrexate, while surgical encompasses procedures to remove the pregnancy tissue such as uterine wedge resection or hysterectomy. However, the management pathway will vary depending on location, extend of myometrial involvement, gestational age at diagnosis, viability, and the patient’s desire to conserve the pregnancy and wishes for future fertility.^5^

## Case Description

A 34-year-old multiparous woman re-presented for review with vaginal discharge and pain in the right iliac fossa on a background of a positive β-HCG. She had been reviewed 1 year previously in the gynecological outpatient clinic for opinion about an incidental finding of a benign asymptomatic fibroid discovered on a pelvic ultrasound performed by her local doctor for investigation for gastric symptoms. Ultrasonography performed with her local doctor revealed a 63 × 60 × 56 mm intramural fibroid in the right lateral posterior uterine wall and a smaller 58 × 30 × 19 mm fibroid adjacent to the external cervical os. Despite extensive counselling against surgical management, the patient underwent an open myomectomy privately.

**Figure 1.**
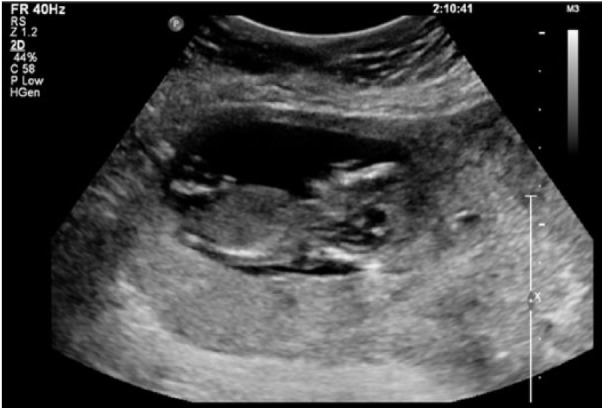
Transvaginal ultrasound with the live intramural pregnancy seen.

**Figure 2.**
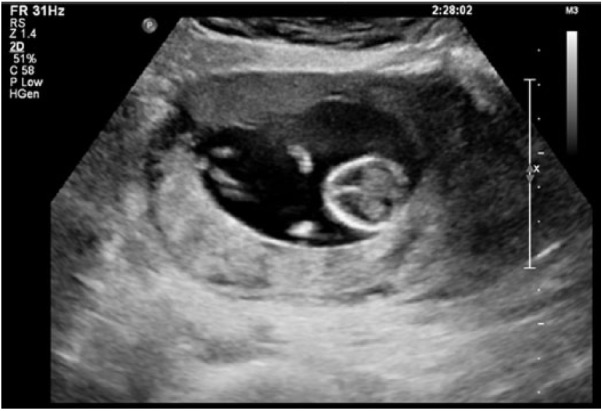
Transvaginal ultrasound with the live intramural pregnancy seen.

**Figure 3.**
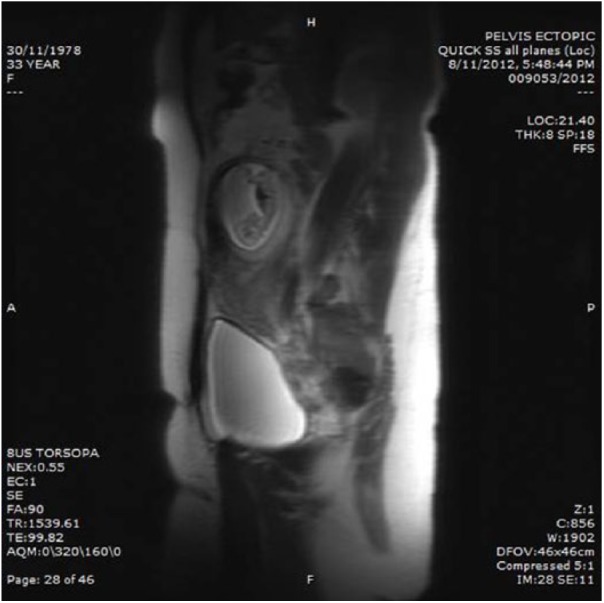
Sagittal views on magnetic resonance imaging of the abdomen demonstrating an intramural pregnancy.

She re-presented 1 year post open myomectomy with vaginal discharge and pain in the right iliac fossa with a 12-week pregnancy by her last menstrual cycle. This pregnancy was spontaneously conceived, and her past obstetric history included 2 normal vaginal deliveries. On review, she was clinically well and a transvaginal ultrasound was performed, which revealed a live intramural ectopic pregnancy, with a thin 3-mm layer of myometrium surrounding the pregnancy (Figures 1 and 2). Placental invasion was also seen, thought to be over the previous myomectomy site. An MRI was performed following the ultrasound to help aid management and determine if fertility sparing intervention options could be considered. MRI revealed a gestational sac (8.0 × 7.9 × 7.0 cm) containing a mobile fetus within the myometrium of the right uterine cornua, with marked thinning of the overlying myometrium to 3 mm, with no clinical features of hemoperitoneum (Figure 3).

Initial management options that were considered included medical management with intra-sac and multidose methotrexate, uterine wedge resection, or hysterectomy. The patient’s desires to conserve fertility were considered, and hence, all conservative management options were explored at multidisciplinary clinical meetings. Subspecialty experts in gynecological surgery and ultrasound were involved in this clinical decision-making process. Unfortunately, medical management with intra-sac and multidose methotrexate was deemed inappropriate due to the advanced gestation age of the pregnancy. Wedge resection of the uterus was also excluded as a viable management option as the location and size of the intramural ectopic pregnancy would result in a large amount of uterine tissue needing to be excised. Senior clinicians, together with the patient, made a uniform decision that it would be safest to proceed with hysterectomy.

A midline laparotomy, total abdominal hysterectomy, and bilateral salpingectomy was performed. Blood loss was minimal, and the patient remained well postoperatively. She was discharged home 3 days later after an uneventful recovery.

## Discussion

This case leaves open for discussion many issues associated with care in women of reproductive age. The patient underwent surgical management of a benign asymptomatic fibroid in the year prior, which increased her risk of future complications, including ectopic pregnancy, placental adhesive disorders, and uterine dehiscence in future pregnancies.^6^ Surgical management of benign asymptomatic fibroids is controversial, with the general consensus being against surgery if patients are asymptomatic.^7^

A review of the literature demonstrates less than 30 published cases of intramural ectopic pregnancy of various etiologies. Cases associated with previous myomectomy specifically are even more uncommon. Bannon et al^6^ described a similar case to the one here. The patient presented at 6 weeks gestation, having undergone an open myomectomy 3 years previously. She was diagnosed with a missed abortion and underwent suction dilatation and curettage. The pathology revealed decidua with foci of necrosis and portions of gestational endometrium, but no placental villi was identified. A subsequent transvaginal ultrasound and computed tomography scan were performed, with an intramural pregnancy diagnosed at the site of the previous myomectomy scar. A single dose of systemic methotrexate was administered; however, a 5-cm avascular intramural pregnancy with possible fistulous tract persisted, and the patient subsequently underwent laparoscopic removal of the intramural pregnancy. In this case, the incorrect initial diagnosis of missed abortion complicated the clinical timeline and delayed the diagnosis. It is important to recognize that intramural pregnancy is often difficult to distinguish from other pathologies; however, performing ultrasonography together with MRI may assist in making an accurate diagnosis and exclude other diagnostic probabilities.^7^

As described, surgical procedures such as myomectomy, salpingectomy, hysteroscopy, and dilatation and curettage are all thought to contribute to the risk of intramural implantation.^5^ Other predisposing factors include assisted reproductive technologies and adenomyosis.^8^ Intramural pregnancy often presents with nonspecific clinical symptoms, including mild vaginal bleeding and abdominal pain; however, some patients may be asymptomatic. Early diagnosis is key in preventing complications, including uterine rupture. Failure to diagnose an intramural pregnancy can result in catastrophic hemorrhage due to the proximity of the gestational sac to the intramyometrial arcuate vasculature.^9^

The pathophysiology of intramural pregnancy is not entirely clear and many hypotheses exist. Previous uterine surgery may lead to the formation of myometrial defects and facilitate intramural implantation.^5^ It is thought that the embryo implants into the myometrium through a microscopic fistula, created through previous uterine surgery, like myomectomy but also as a consequence of previous caesarean section.^10^ In a similar way, the embryo may implant, together with endometrial tissue, into the myometrium during the development of adenomyosis.^11^ Furthermore, artificial implantation of the embryo during assisted reproductive technologies may also result in development of an intramural pregnancy.^2^ The myometrial defect potentially created from these procedures is thought to allow trophoblast invasion into the myometrium, which may enable intramural implantation.^5^

Transvaginal ultrasound is considered the first-line imaging technique for diagnosis of ectopic pregnancy,^7^ with a diagnostic accuracy of 90.9%.^12^ The other imaging modality alternatively used, MRI, has a diagnostic accuracy of 96%.^13^ This case report utilized both imaging techniques as diagnostic tools and for surgical planning, with both playing an important role in constructing the overall clinical picture. In cases of unusual or rare pregnancies, the use of such diagnostic tools early in the gestation has allowed management to shift predominately from radical surgical management, to more conservative, minimally invasive interventions.^14^

In patients who present clinically well, without signs of hypovolemic shock with suspected uterine rupture, medical or surgical management options can be considered. A recent study by Ramkrishna et al^13^ has shown that the use of systemic methotrexate and or local intra-sac methotrexate (with intra-sac KCl if embryonic heart activity is present) is a successful intervention for management of nontubal ectopic pregnancies, especially in those women wishing to preserve fertility. If diagnosis is made at an early gestation, prior to rupture, conservative options can be considered.^15^ The median gestational age of successful medical management within the study by Ramkrishna et al^13^ in all ectopic pregnancy sites was less than 8 weeks gestation. Medical management can be considered at early gestations in cases when the patient is clinically stable. Given the advanced gestational age and presence of a fetal heartbeat in this case, the use of systemic or local injection of methotrexate did not seem appropriate, and hence, surgical management was required. Surgical options can include excision of the intramural pregnancy or definitive hysterectomy, these can be done laparoscopically or open.^16^

This report adds to the literature and explores some of the diagnostic and management challenges uncommon ectopic implantation sites can pose. Women often present with nonspecific clinical symptoms, which makes diagnosis difficult. Previous uterine surgical procedures have been shown to increase the risk of fertility complications, including intramural pregnancy, and as such, patients with known risk factors should seek medical attention early in their pregnancy. Transvaginal ultrasound plays a pivotal role, with MRI also adding to diagnostic accuracy. Overall, diagnosis and treatment should be tailored to individual patient factors, with multidisciplinary team management playing a pivotal role.
